# Supplementary material for: The impact of physical exercise on university students’ life satisfaction: The chain mediation effects of general self-efficacy and health literacy
Source: PLoS One. 2025 Jun 9;20(6):e0325835. doi: 10.1371/journal.pone.0325835 (PMC12148183; doi:10.1371/journal.pone.0325835)
Supplement: Attachment — Submitted filename: Response to Reviewer.docx [file pone.0325835.s003.docx]

Title： The Impact of Physical Exercise on University Students' Life Satisfaction: The Chain Mediation Effects of General Self-Efficacy and Health Literacy

Type： Research

Journal： PLOS One

Submission ID： PONE-D-25-08597R1

Thank you very much for your comments and professional advice. These opinions help to improve the academic rigor of our manuscript. Based on your suggestion and request, we have made corrected modifications to the revised manuscript. Here are point-by-point responses to your comments. We hope that our work can be improved again. Furthermore, we would like to show the details as follows:

Sincerely,

On behalf of all authors

**Point-by-Point Responses to Reviewer 1**

**Comment #1:**

**The data collection period mentioned in line no. 161 (Nov 2023) predates the recruitment period in line no. 160 (Aug–Sep 2024), which appears inconsistent and should be clarified.**

**Response# 1:**

Thank you very much for pointing this out. We have revised the text to address the inconsistency, and the details are in lines 159–161 of the manuscript.

**Comment #2:**

**The decision to dichotomize general self-efficacy (low/normal) is mentioned under variable measurement in line no. 208, but not reflected in later analyses. Was this grouping used at any point? If not, consider removing the detail to avoid confusion.**

**Response# 2:**

Thank you very much for your insightful comment. We have made the necessary revisions to ensure consistency throughout the manuscript. The details of the changes are in line 207. We appreciate your attention to this matter.

**Comment #3:**

**The conclusion that general self-efficacy and health literacy fully mediate the relationship between exercise and life satisfaction in line no. 398 and 441 should be framed with caution. While statistically accurate, the magnitude of the total effect (0.045) is small and should not be overstated.**

**Response# 3:**

Thank you for your feedback. We have revised the conclusion and highlighted the changes in lines 398–404 and 439–447. We now present the findings more cautiously, considering the small total effect size.

**Comment #4:**

**Although all references are numbered in order of appearance and in-text citations correspond to the reference list correctly., but some journal names are abbreviated like J Behav Med, some article titles were in capitals and DOIs are missing in most of the references. It is suggested to align the referencing style with PLOS ONE referencing style.**

**Response# 4:**

Thank you for your detailed feedback on the referencing style. We have thoroughly revised the reference list to align with the PLOS ONE style. This includes standardizing journal names, formatting article titles correctly, and ensuring that all DOIs are included. The changes can be found in the reference section of the manuscript.

**Comment #5:**

**The mediation analysis reveals that general self-efficacy accounts for 79.5% of the total indirect effect, while the chain mediation pathway (via both general self-efficacy and health literacy) accounts for 11.4%. You are encouraged to highlight why general self-efficacy appears to be a more potent mediator and elaborate on potential theoretical underpinnings for this finding.**

**Response# 5:**

Thank you for your feedback. We have revised the manuscript and elaborated on why general self-efficacy is a more potent mediator in lines 311–322. The discussion now includes theoretical underpinnings based on Bandura's Social Cognitive Theory.

**Comment #6:**

**Expand on why the direct effect of physical exercise becomes nonsignificant—e.g., are there competing pathways not captured in the model?**

**Response# 6:**

Thank you for your suggestion. I have expanded the explanation in the manuscript regarding why the direct effect of physical exercise becomes nonsignificant. Specifically, I have added a discussion on potential competing pathways that may not be captured in the current model. The details can be found in the " Methods " section.

**Comment #7:**

**While demographic variables (e.g., gender, ethnicity, grade) were included as covariates, no moderation analysis was conducted. Given the large sample and observed gender differences in physical activity (η² = 0.155), you are encouraged to consider whether the mediation model operates differently across subgroups (e.g., males vs. females), either in this paper or as a future direction.**

**Response# 7:**

Thank you for the reminder. We treated gender, Ethnicity, and grade solely as covariates to control for potential confounding factors in the mediation analysis.

**Comment #8:**

**The final paragraphs of the discussion somewhat repeat earlier points; consider tightening the conclusion.**

**Response# 8:**

Thank you for your suggestion. We have revised the final paragraphs of the discussion to avoid repetition and tighten the conclusion. The changes can be seen in the last paragraph of the discussion section.

**Comment #9:**

**In Table 2, clarify the layout to improve readability across demographic variables and their statistical outcomes.**

**Response# 9:**

Thank you for your suggestion. We have revised the layout of Table 2 to enhance its readability, ensuring clearer presentation of demographic variables and their corresponding statistical outcomes. The updated table is now included in the manuscript.

**Point-by-Point Responses to Reviewer 2**

**Comment #1:**

**In line no. 174, the explanation is required for "no ethical committee waived the need for consent".**

**Response# 1:**

Thank you for your comment. In line 174, we have added an explanation to clarify that the study did not require ethical committee approval due to its nature as a secondary analysis of anonymized data. The details can be found in the revised manuscript.

**Comment #2:**

**Reliability of SWLS is not provided.**

**Response# 2:**

Thank you for pointing this out. We have now included the reliability information for the SWLS in lines 207–211 of the manuscript.

**Comment #3:**

**For lines 68-72, a reference to data related to challenges faced by university students needs to be provided.**

**Response# 3:**

Thank you for your valuable feedback. We have incorporated an appropriate reference to substantiate the data regarding the challenges faced by university students, as indicated in lines 68–72.
